# The development of the H3 Package: a Package of High-Priority Health Services for Humanitarian Response

**DOI:** 10.1136/bmjgh-2025-020120

**Published:** 2026-01-16

**Authors:** Andre Griekspoor, Vinay N Kampalath, Morgan C Broccoli, John Fogarty, Eba Pasha, Nureyan Zunong, Karl Blanchet, Teri Reynolds

**Affiliations:** 1 Department of Health Emergency Interventions, World Health Organization, Geneva, Switzerland; 2 Geneva Centre of Humanitarian Studies, University of Geneva, Geneva, Switzerland; 3 Department of Integrated Health Services, World Health Organization, Geneva, Switzerland; 4 Global Health Cluster, World Health Organization, Geneva, Switzerland; 5 Save the Children, Washington, DC, USA; 6 Geneva Centre of Humanitarian Studies, Faculty of Medicine, University of Geneva, Geneva, Switzerland

**Keywords:** Delivery of Health Care, Global Health, Health systems, Health Services Accessibility, Universal Health Care

## Abstract

**Introduction:**

Humanitarian crises substantially impact the health of affected populations, and the scale of humanitarian need is at a historic high level. To more effectively support the growing number of people affected by humanitarian crises, the WHO, the Global Health Cluster and humanitarian partners undertook an initiative to define a core set of services to be delivered during a humanitarian response. This paper describes that process.

**Methods:**

The methodology used in the development of a Package of High-Priority Health Services for Humanitarian Response (the H3 Package) was derived from an evidence-informed deliberative process and included the following steps: identifying operational assumptions, defining the burden of disease context, identifying services in relevant existing service packages, identifying priority-setting criteria, defining service delivery platforms, selecting services based on WHO’s Universal Health Coverage Compendium of Health Interventions services and conducting an expert validation process.

**Results:**

The final H3 Package is organised across six domains: foundations of care, sexual and reproductive health, violence and injury, rehabilitation and palliative care, communicable diseases, and non-communicable diseases and mental health. The full package is available online via the WHO Service Planning, Delivery and Implementation Platform. The H3 Package is intended as a reference to be contextualised, and steps for contextualisation are proposed.

**Conclusion:**

The H3 Package sets a global standard for a core set of health services that humanitarian actors can reasonably be expected to deliver in humanitarian settings. This paper provides an overview of the H3 package, describes the methods used in its development and suggests steps for package contextualisation and implementation.

**WHAT IS ALREADY KNOWN ON THIS TOPIC**

- Given the increasing scale and impact of humanitarian crises, evidence-informed approaches to health service provision are needed in humanitarian settings to help achieve universal health coverage (UHC).
- Packages of priority health services are an important tool that countries use to achieve UHC and have been described in several humanitarian contexts.
- Although national service packages have been developed in several conflict-affected countries (Afghanistan, Somalia, Yemen), there has yet to be a global effort to define an integrated package of priority health services for humanitarian response.

**WHAT THIS STUDY ADDS**

- In this study, we describe the development of the H3 Package, the first global reference Package of High-Priority Health Services for Humanitarian Response.
- This reference package notably includes basic non-communicable disease, rehabilitative and palliative care services, which are increasingly important in humanitarian contexts.
- We also provide a blueprint for implementation and adaptation of the H3 Package to local contexts and recommend a series of steps and priority-setting criteria to accomplish this in a transparent manner.

**HOW THIS STUDY MIGHT AFFECT RESEARCH, PRACTICE OR POLICY**

- By defining the H3 Package, we apply the principles of UHC to a humanitarian context and create a foundation to develop future packages of priority health services in humanitarian settings.
- The H3 Package identifies a set of prioritised health services that could be feasibly implemented, increases accountability to affected populations and identifies operational entry points for the Humanitarian–Development Nexus.

## Introduction

 Over the last decade, the worldwide burden of humanitarian crises has more than doubled, with 339 million people in need of humanitarian assistance in 2023.^1^ Humanitarian crises have also become increasingly protracted, reflecting complex political and contextual circumstances that have produced high levels of displacement.^2^ By 2030, it is estimated that more than half of those living in extreme poverty worldwide will live in fragile, conflict-affected and violent settings.^3^ To more effectively support the growing number of people affected by humanitarian crises, WHO, the Global Health Cluster (GHC) and several humanitarian partners and academics undertook an initiative to define a core set of services to be delivered during a humanitarian response; this process culminated in the development of a Package of High-Priority Health Services for Humanitarian Response (the H3 Package), which was published by WHO in July 2024.^4^ This paper describes the methods used to develop the H3 Package.

People living in humanitarian settings are at an increased risk of numerous health threats including infectious diseases, malnutrition, gender-based violence and mental and psychosocial stressors.^5^ 60% of preventable maternal deaths, 53% of deaths in children younger than 5 years and 45% of neonatal deaths take place in fragile settings of conflict, displacement and natural disasters.^6^ The burden of non-communicable diseases (NCDs) has also been increasing in humanitarian settings.^7^ Health development partners and the humanitarian sector face numerous challenges in effectively providing services to people affected by these crises and, with limited resources and many competing pressures, health actors in low-income and middle-income countries (LMICs) must make difficult decisions about which health services to deliver. The GHC, WHO and humanitarian partners have previously developed guidance on why and how to develop context-specific health service packages, but these guidance documents have not been consistently implemented.^5 8^

Although national service packages have been developed in several conflict-affected countries (Afghanistan, Somalia, Yemen), there has yet to be a global effort to define an integrated package of priority health services for humanitarian response.^9 10^ The H3 Package was designed to apply the principles of universal health coverage (UHC) to a humanitarian context and define a set of prioritised health interventions that could feasibly be delivered to populations affected by humanitarian crises during protracted emergencies.^11 12^ The H3 Package was also developed to promote accountability of humanitarian partners to affected populations by ensuring necessary services are provided to all and identifying opportunities to facilitate an operational entry point for the Humanitarian–Development Nexus by harmonising humanitarian packages with national packages. The H3 Package can be easily linked with national service packages, but it is not intended to define or replace a national package of priority health services.

## Development of the H3 Package

The methodology used in developing the H3 Package was derived from an evidence-informed deliberative process for priority setting and involved a situational analysis, criteria identification, evidence collection and generation of recommendations by the GHC Essential Health Services (EHS) Core Group.^4 13^

A multistep methodology was undertaken that included:

- Identifying operational assumptions.
- Defining the burden of disease (BoD) context.
- Identifying services in relevant existing service packages.
- Identifying priority-setting criteria.
- Defining service delivery platforms.
- Developing a zero-draft of the package.
- Conducting an expert validation process.

### Identifying operational assumptions

Humanitarian crises were defined as events in a country or region that cause serious disruption to the functioning of a society and result in human, material or environmental losses which exceed the ability of affected people to cope using their own resources.^14^ Given the prolonged nature of many modern-day humanitarian crises, the H3 Package was specifically designed for protracted emergencies or for insecure environments in which a significant proportion of the population is acutely vulnerable to death, disease and disruption of livelihoods over a prolonged period.^15^

The H3 Package was designed to reflect core services to be provided and/or supported by humanitarian actors. The GHC EHS Core Group developed the following operational assumptions^4^:

- National health systems have limited resources (e.g., workforce, medicines, health information systems).
- The health workforce has been adequately trained to provide basic services that address high-burden diseases.
- Humanitarian partners are coordinated under activated interagency standing committee protocols to support national response capacity.^16^
- Supply chains are supported by humanitarian partners and can provide necessary medical supplies to facilities.
- Gaps in service delivery within a local health system can be addressed.

These operational assumptions provided the foundation for a shared understanding of the context guiding the design of the H3 Package; however, they did not serve as the priority-setting criteria for selecting services, which were determined in a subsequent stage.

### Defining the BoD context

The leading causes of death and disability in humanitarian settings were estimated using BoD data from fourteen countries with active or recent humanitarian crises (Afghanistan, Bangladesh, Colombia, the Democratic Republic of Congo, Ethiopia, Iraq, Mali, Nigeria, Pakistan, the occupied Palestinian territories, Somalia, South Sudan, the Syrian Arab Republic and Yemen). The top 10 causes of death and disability were identified for each selected country using the Institute of Health Metrics and Evaluation’s Global Burden of Disease (GBD) 2019 study.^17^

The national BoD profiles from the selected countries showed high disease burdens from respiratory tract infections, diarrhoeal diseases and neonatal and congenital disorders. Stroke and ischaemic cardiac disease were among the top three causes of morbidity and mortality in seven countries, and an analysis of BoD trends in all selected countries revealed a recent rise in the prevalence of these diseases.^18^ Diabetes had a high burden in six countries, and other infectious diseases such as malaria and meningitis were particularly prevalent in African countries. Injuries, including road traffic injuries, interpersonal violence and conflict, were common in seven countries.^4^

As the GBD data did not provide subnational data for humanitarian contexts, an additional review was conducted to identify causes of morbidity and mortality in humanitarian settings not observed in national-level data. Data were pulled from significant humanitarian crises occurring at the time of analysis, where health clusters were activated and open-source data were available. Country field reports and health cluster bulletins from Afghanistan, Bangladesh (Cox’s Bazar), the Democratic Republic of Congo, Ethiopia (Tigray), northern Nigeria, the occupied Palestinian territories, Somalia, South Sudan, the Syrian Arab Republic and Yemen were reviewed and analysed. In these contexts, communicable diseases were still relevant, and vaccine-preventable diseases such as measles were more frequently mentioned. Malnutrition, mental illness and sexual and reproductive health (SRH) disorders were also frequently cited as high-burden diseases. The BoD lists at the national and humanitarian levels were collated to identify diseases that required similar services, and diseases were reviewed to determine relative frequency in these contexts.^4^

High-burden diseases, injuries and other health threats in the selected LMICs and subnational humanitarian contexts were used as a reference for the prioritisation of services (box 1).

Box 1 **High-burden diseases, injuries and other health threats frequently identified in humanitarian settings^4^**

Cardiovascular diseases (including ischaemic heart disease, cerebrovascular disease and hypertension)

Depressive and anxiety disorders

Diabetes

Diarrhoeal diseases

Lower respiratory tract infections

Malaria

Measles

Meningitis

Neonatal and congenital disorders

Nutritional disorders (including protein-energy malnutrition and micronutrient deficiencies)

Sexual and reproductive health disorders

Trauma (including road injuries, conflict and violence)

### Identifying services in relevant existing service packages

Services from existing health service packages used in countries or areas with humanitarian contexts were reviewed to identify domains of care and high-priority services for the H3 Package. Health service packages from Afghanistan, northwest Syrian Arab Republic, Somalia, South Sudan and Yemen were chosen based on input from country health clusters and humanitarian partners as they represented diverse contexts and were, at the time of analysis, among the areas with the highest humanitarian need.

Services were also identified from the Disease Control Priorities High Priority Package (DCP3 HPP) and the Minimum Initial Service Package (MISP) for SRH in crisis situations.^19^,^21^ All services were mapped to WHO’s Service Planning, Delivery and Implementation (SPDI) platform based on the UHC Compendium of Health Interventions, a comprehensive database of health services designed to support countries in making progress toward UHC. The UHC Compendium contains more than 3500 health services and provides a structured architecture on which to build a health service package. Through the SPDI platform, services in the UHC Compendium are linked to health workforce and product data to support service delivery implementation. Services described in the five country packages, the DCP3 HPP and the MISP had substantial differences in the detail of the services, and the standardised structure in the UHC Compendium was utilised to harmonise those variations.

The services described in the seven reference packages primarily addressed the high-burden diseases found in the analysis and included services for communicable diseases, NCDs, mental health, nutrition and injury. All reviewed packages included core services for SRH. However, few services for NCDs were included in these packages, despite evidence of their growing importance in countries affected by protracted conflict.^18^ Using the analyses from BoD data and the seven reference packages, six clinical domains were identified: foundations of care, SRH, violence and injury, rehabilitation and palliative care, NCDs and mental health, and communicable diseases. Cross-cutting services necessary to support people with emergency or undifferentiated presentations were included and grouped into the foundations of care domain.

### Identifying priority-setting criteria

The H3 Package was designed to define the core services to be provided or supported by the humanitarian response where national delivery capacities are disrupted. Six criteria were selected to prioritise interventions as core services (table 1); the GHC EHS Core Group adapted these criteria from existing priority-setting standards.^5 8 22^ Local feasibility was added to prioritise interventions for which no extensive additional training was required. Services that address the health needs of vulnerable communities affected by the conflict or human rights violations (eg, clinical management of survivors of rape) were also included, even when services did not address high-burden diseases.

**Table 1:** Principal priority-setting criteria for core services in the H3 Package

Impact on morbidity and mortality	Evidence-based and time-sensitive services that have the greatest potential to decrease morbidity and mortality
Affordability	Low-cost and high-impact services
Feasibility	Local capacity of the healthcare system to deliver the service
Equity	Equal treatment for equal need, including all people targeted by the humanitarian response
Effectiveness	Specific services that are required to manage a particular condition and without which would result in ineffective care
Humanitarian imperative	Services that address health needs that arise from conflict or violations of human rights, including interventions that help people recover from harm or alleviate suffering

In line with the first criterion, services that address the initial approach to common signs and symptoms prior to a definitive diagnosis were included. Criteria 2, 3 and 5 informed the exclusion of complex services requiring substantial financial inputs or infrastructure, such as curative chemotherapy for cancer. Using criterion 3, resource requirements were reviewed so that selected services could be implemented using medicines and equipment included in standard humanitarian medical kits. Criterion 6 informed the inclusion of services for people harmed by gender-based violence as well as palliative and rehabilitation services to alleviate suffering.

When a service was considered valuable but did not meet the criteria for a core service, it was included as an ‘extended service’. These services are to be considered for inclusion if and when the operational capacities of local and humanitarian partners allow.

### Defining service delivery platforms

While health systems vary, five default levels of service delivery were defined as a starting point for priority setting using the H3 Package: community-based services, outpatient services, dedicated pregnancy/birth care facilities, first-level referral hospitals and second-level referral hospitals. Service delivery levels were defined based on typical contextual circumstances in the field. Details on the default delivery platforms are provided in table 2.

**Table 2:** Definitions of service delivery platform

	Workforce and services	Medicines and diagnostics
Community	Community health worker with 3–6 months of basic training	Very limited medications
Outpatient	Nurse performing consultations A skilled birth attendant or midwife may be on-call A medical doctor is not routinely available Referral to first-level referral hospital by private transport within 2 hours	Oral medications and a limited complement of intramuscular medications Limited laboratory and imaging capabilities
Dedicated pregnancy/birth care facility	Skilled birth attendant or midwife providing a complement of Basic Emergency Obstetric and Newborn Care services available 24/7	Relevant obstetric parenteral medications available Limited laboratory and imaging capabilities
First-level referral hospital	Medical doctor with knowledge of non-communicable diseases and mental and psychosocial health Basic operative care available Comprehensive Emergency Obstetric and Newborn Care services available 24/7	Oral and parenteral medications Basic laboratory and imaging capabilities
Second-level referral hospital	Medical doctors with subspecialty training Subspecialty care, including operative care	More complete complement of oral and parenteral medications More advanced laboratory and imaging capabilities

### Developing a zero draft of the package

The authors of this manuscript defined an initial candidate package using the structured architecture from WHO’s UHC Compendium of Health Interventions, and services were selected to address the high-burden diseases identified in humanitarian contexts. The authors then compared the list of services to high-priority services across the reference packages and conducted an initial review to assess for face and content validity. Country and health cluster representatives from the Syrian Arab Republic, Yemen and Somalia, selected for their experience in the field and with health packages, were included in a second round of reviews that refined the package further using a modified Delphi approach.^23^ The authors and country and cluster partners independently reviewed each service in the draft list and designated them as core, extended or excluded from the package using the priority-setting criteria defined in table 1. When services were identified as core or extended, they were also assigned a minimum feasible level of service delivery. This group met virtually to discuss actions that did not meet a threshold of 80% agreement and a decision was adopted after a majority of committee members agreed. This process yielded an initial zero draft of the package.

### Conducting an expert validation process

The draft package was subsequently reviewed by panels of relevant content experts for each of the domains using a similar modified Delphi approach. Content experts were selected for their expertise both in clinical work and experience working in humanitarian or low-resource settings. Content experts included those working in the field as well as regional and global headquarters, and emphasised diversity in gender and global representation (see online supplemental tables for content expert panel characteristics).

Content experts independently reviewed each service and designation according to the same priority-setting criteria defined in table 1. Each expert panel met virtually to discuss actions that did not meet a threshold of 80% agreement, and a decision was adopted after a majority of experts agreed. During the expert review, a few organisational changes were made: the mental health domain was expanded to include disorders of substance use and epilepsy to align with WHO’s Intervention Guide for mental, neurological and substance use disorders.^24^ Rehabilitation and palliative care services were added as distinct domains. Actions were then aligned with the contents of emergency health kits. The manuscript authors completed the final review, integrating experts’ consensus recommendations and providing the final votes when consensus was not achieved. The authors then reviewed the entire package to ensure harmonisation of the package across domains.

### Ethics

No ethical review was required for this study as it only involved a literature review and gathering expert consensus to generate a list of high-priority interventions. Patients, affected populations and the public were not involved in the design, conduct, reporting or dissemination of this research.

## Contents of the H3 Package

The final H3 Package is organised across six domains as shown in table 3. Services are assigned to the lowest level facility where they could feasibly be expected to be delivered by humanitarian partners.^4^ Notably, the H3 Package includes basic services for NCD, palliative and rehabilitative care, which have previously been neglected in humanitarian contexts.

**Table 3:** Overview of H3 Package contents by domain*

Domain	Intervention categories
Foundations of care	First access, continuity and coordination of care Approach to emergency conditions Approach to common signs and symptoms Nutrition
Sexual and reproductive health	Antenatal and postnatal care Labour and childbirth care Abortion Ectopic pregnancy Sexual health, contraception and family planning Female genital mutilation
Violence and injury	General management of injury Gender-based, intimate partner and sexual violence Mechanical, thermal and chemical injuries Envenomation, poisoning and toxic injuries
Rehabilitation and palliative care	Rehabilitation for musculoskeletal, neurologic and cardiorespiratory systems Rehabilitation for cognition and communication Palliative care
Communicable diseases	Vaccinations HIV Tuberculosis Malaria Lower respiratory infections Diarrhoea Sexually transmitted infections Other common infections (including measles and meningitis)
Non-communicable diseases and mental health	Cardiovascular and respiratory disorders Diabetes Mental health disorders Substance use disorders Other non-communicable diseases

* Adapted from H3 Package guidance.^4^

Using a life course perspective, the H3 Package includes interventions intended to target health conditions at every age and stage of life. While other high-priority packages of care have included child health-specific domains focusing on high burden paediatric diseases (eg, lower respiratory tract infections, diarrhoeal illness, malaria and malnutrition), the H3 Package’s domains categorise interventions based on diseases, injuries or health threats that use clinically similar approaches to management, irrespective of age. This cross-cutting approach recognises that neonates, infants, children and adolescents in humanitarian settings increasingly have diverse care needs, including care for sepsis, trauma, NCDs (eg, asthma and diabetes), sexual health and rehabilitation.

## Contextualising the H3 Package

The H3 Package is intended as a reference to be contextualised, accounting for differences in BoD, baseline service delivery capacities and health system organisation. Settings with higher levels of pre-existing capacity may require an immediate expansion of services beyond those described in the H3 Package. Although the H3 Package was designed as a feasible core, there may be contexts where some components of the package cannot be implemented, such as settings with human and/or material resource constraints. Contextualisation of the H3 Package should include the following steps:

1. *Characterise existing package(s)*. If available, existing service packages delivered as part of the humanitarian response and services delivered during normal system operations should be reviewed and visualised.
2. *Conduct a BoD analysis*. Services that address specific conditions or diseases should be added or removed from the package as appropriate. For example, the H3 Package does not include services for neglected tropical diseases, which may be relevant in some settings. Conversely, other settings may choose to remove services described in the H3 package which are not relevant (eg, malaria).
3. *Map current service delivery*. Technical experts from operational health partners and others involved in direct clinical service delivery should conduct a structured review of all health areas to determine what services are currently being delivered and where they are being delivered. Gaps or bottlenecks in existing service delivery should be noted and highlighted.
4. *Use the H3 Package as a reference and compare it to the current service delivery*. The services currently being delivered should be compared with existing packages, the local BoD and reference standards such as the H3 Package to identify gaps and determine feasibility.
5. *Contextualise and finalise the humanitarian package of services.* Once gaps in service delivery have been identified, the humanitarian package of services should be contextualised. There are several ways that partners may choose to contextualise the H3 Package:

- Core services may be assigned to a lower delivery platform when additional resources are available (services should be aligned with national policies that define the scope of care for health workers).
- Extended services may be included when they are highly relevant and feasible to provide.
- Additional services described in the UHC Compendium or other reference packages may be added to the package, either as core or extended services.
- Services where bottlenecks have been identified should be highlighted.

1. *Prepare for implementation*. A package that is not successfully implemented has no impact, and it is critical to contextualise health worker, product and medicine requirements to support package implementation. Some considerations that support the optimisation of service delivery:

- Review the scope of services and staffing requirements by platform.
- Define health worker competencies based on visits and tasks.
- Identify resource requirements by platform for procurement planning.
- Align protocols for optimal movement across platforms.

A package may also need to be contextualised subnationally based on regional differences in health risks or operational feasibility, such as in areas with insecurity and a high incidence of traumatic injuries.

The H3 package was constructed using WHO’s SPDI platform and UHC Compendium database, which many countries use to formulate national packages of services for UHC. This alignment orients the horizon of convergence between national and humanitarian planning and creates interoperability among humanitarian and national packages of priority health services.

SPDI can assist humanitarian partners in conducting a structured review of existing services, contextualising services using the H3 Package and finalising a humanitarian package (UHCC.who.int/UHCPackages). The SPDI Platform links to human and material resource data, which can be used to define resource requirements and support costing analyses. The H3 Package was intended to be adaptive rather than static, and after the initial creation described in this manuscript, the H3 Package was updated on the SPDI platform to be consistent with the evolving architecture and terminology.

The H3 Package currently does not include a mobile health clinic as a default service delivery platform, though work is being done to add this given their frequent deployment in humanitarian contexts. Should humanitarian actors wish to include mobile health clinics as part of service delivery, the SPDI Platform can support this contextualisation.

Formal costing of the H3 Package has yet to be completed. Only two of the packages we used to inform it were costed, US$6.90 per capita in Afghanistan and US$40 per capita in Northern Syria.^25 26^ The package estimate for Northern Syria may be higher as it included more services for NCDs. Neither package captures the additional operational, political and security costs inherent to humanitarian service delivery. Future costing efforts using tools such as the SPDI Platform will be critical to support context-specific cost analyses and to guide resource mobilisation for implementation.

## Implementing the humanitarian package

A well-designed package helps define models of care by providing detailed information on relevant delivery platforms and associated human and material resource needs, resulting in packages that are realistic and implementable^5^.

Additional analyses and planning that may be considered by local partners to further support implementation include:

1. A cost analysis of the package, which can guide adjustments to align with available budgets and ensure that service users are not required to pay user fees.^27^
2. A feasibility analysis, which can help determine the ability of the health system to deliver services and identify anticipated bottlenecks. The mapping exercise described above can provide a strong foundation for a feasibility analysis.
3. A monitoring and evaluation system, which will enhance implementation and preferably should be integrated with the Health Management Information System.^6^
4. A strategic and operational plan that describes the required numbers and mix of service delivery platforms using a district health system approach. The plan should consider the security and operational context of different areas, variations in population density and the ability to refer to functional public health facilities.

The humanitarian package should be complemented by other quality domains, such as diagnostic and treatment protocols, quality improvement processes, facility management capacities and the establishment of appropriate engagement with communities, including independent complaint mechanisms. The target population should always be consulted to better understand demand-side barriers. Preliminary guidance for the adaptation and implementation of the H3 Package has been developed and describes these steps in further detail.^4^

## Limitations

The use of a predefined humanitarian package based on BoD for low-resource settings creates a risk for both under-prioritisation and over-prioritisation of some conditions. By using a variety of countries in the BoD analysis, however, we believe we have adequately captured the breadth of conditions in humanitarian settings. Even though we made efforts to define a realistic package by including humanitarian professionals and clinicians with extensive and varied field experiences, the threshold for consensus was not always met to classify a service as core or extended, or when assigning a service to the lowest feasible level of service delivery. The final selection of some services and their service delivery classifications was based on judgement, and in some cases may have been aspirational. Furthermore, this effort did not undertake a comprehensive economic evaluation of the services described in the package. Nonetheless, as we used packages with low costs per capita as references, we believe the H3 Package provides an affordable starting point for countries tailoring their packages. Finally, this effort did not include beneficiary groups in the development of this package. We did, however, consider priority-setting criteria that have been shown to have high beneficiary acceptability, such as impact on morbidity and mortality, equity and criteria for a patient-centred approach.^28^ Beneficiary needs and acceptability should be integrated into any country-level contextualisation and adaptation process.

## Conclusion

Humanitarian crises substantially impact the health of affected populations, and the scale of humanitarian need is at a historic high level. The H3 Package sets a global standard for a core set of health services that humanitarian actors can reasonably be expected to deliver in humanitarian settings and is intended as a reference to be contextualised. The H3 Package is now undergoing pilot implementation with country partners in several humanitarian contexts, and feasibility and implementation analyses are being conducted. These packages will be costed and used to determine realistic budgets in close collaboration with donors supporting these contexts.

A priority package of health services is powerful. It can define benefits for affected communities, ensure accountability for implementing partners, create a foundation for service planning and inform resource requirements for staffing and medical supplies. Done well, a humanitarian package supports models of care that can adapt to changes in the BoD, guide formal and in-service training of health workers against national standards, and align facility standards with quality and patient safety processes. This process creates an operational entry point for putting the Humanitarian–Development Nexus into practice by harmonising a humanitarian package with a national package. Finally, it makes an explicit contribution to UHC for communities that are otherwise excluded.

## Supplementary material

10.1136/bmjgh-2025-020120 online supplemental file 1

## Data Availability

Data are available upon reasonable request.
