# Supplementary material for: The development of the H3 Package: a Package of High-Priority Health Services for Humanitarian Response
Source: BMJ Glob Health. 2026 Jan 16;11(1):e020120. doi: 10.1136/bmjgh-2025-020120 (PMC12815147; doi:10.1136/bmjgh-2025-020120)
Supplement: online supplemental file 1 [file bmjgh-11-1-s001.pdf]

Supplemental Tables: Composition of content expert panels

**Sexual and reproductive health**	
Female:	9
Male:	1
Total:	10
Organizations Represented	
WHO	5
UNFPA	1
IAWG	1
MSI Reproductive Choices	1
UNHCR	1
Independent expert	2

**Nutrition**	
Female:	3
Male:	4
Total:	7
Organizations Represented	
MSF	1
Aga Khan University	1
Save the Children	2
UNICEF	1
UNHCR	1
Action Contre la Faim	1

**Mental health**	
Female:	4
Male:	6
Total:	10
Organizations Represented	
WHO	3
PAHO	1
UNICEF	1
MSF	1
LSHTM	1
Queen Margaret University	1
Independent expert	2

**NCDs**	
Female:	6
Male:	6
Total:	12
Organizations Represented	
WHO	5
LSHTM	1
MSF	1
ICRC	1
IRC	1
Save the Children	1
UNHCR	1
University of Cape Town	1

**Communicable diseases**	
Female:	7
Male:	1
Total:	8
Organizations Represented	
WHO	1
Johns Hopkins University	1
IRC	3
George Washington University	1
Medical Teams International	1
UNHCR	1

**Violence and injury**	
Female:	2
Male:	6
Total:	8
Organizations Represented	
WHO	3
Karolinska Institut	1
Save the Children	1
MSF	2
University of Geneva	1
